# Supplementary material for: OPTIMIR, a novel algorithm for integrating available genome-wide genotype data into miRNA sequence alignment analysis
Source: RNA. 2019 Jun;25(6):657–68. doi: 10.1261/rna.069708.118 (PMC6521604; doi:10.1261/rna.069708.118)
Supplement: Supplemental Material [file supp_25_6_657__index.html]

OPTIMIR, a novel algorithm for integrating available genome-wide genotype data into miRNA sequence alignment analysis — Supplemental Material 

# OPTIMIR, a novel algorithm for integrating available genome-wide genotype data into miRNA sequence alignment analysis

## Supplemental Material

- Supplemental\_Fig\_S1.docx
- Supplemental\_Fig\_S2.docx
- Supplemental\_Table\_S1.xlsx
- Supplemental\_Table\_S2.xlsx
- Supplemental\_Table\_S3.xlsx
- Supplemental\_Table\_S4.xlsx
