# Supplementary material for: OPTIMIR, a novel algorithm for integrating available genome-wide genotype data into miRNA sequence alignment analysis
Source: RNA. 2019 Jun;25(6):657–68. doi: 10.1261/rna.069708.118 (PMC6521604; doi:10.1261/rna.069708.118)

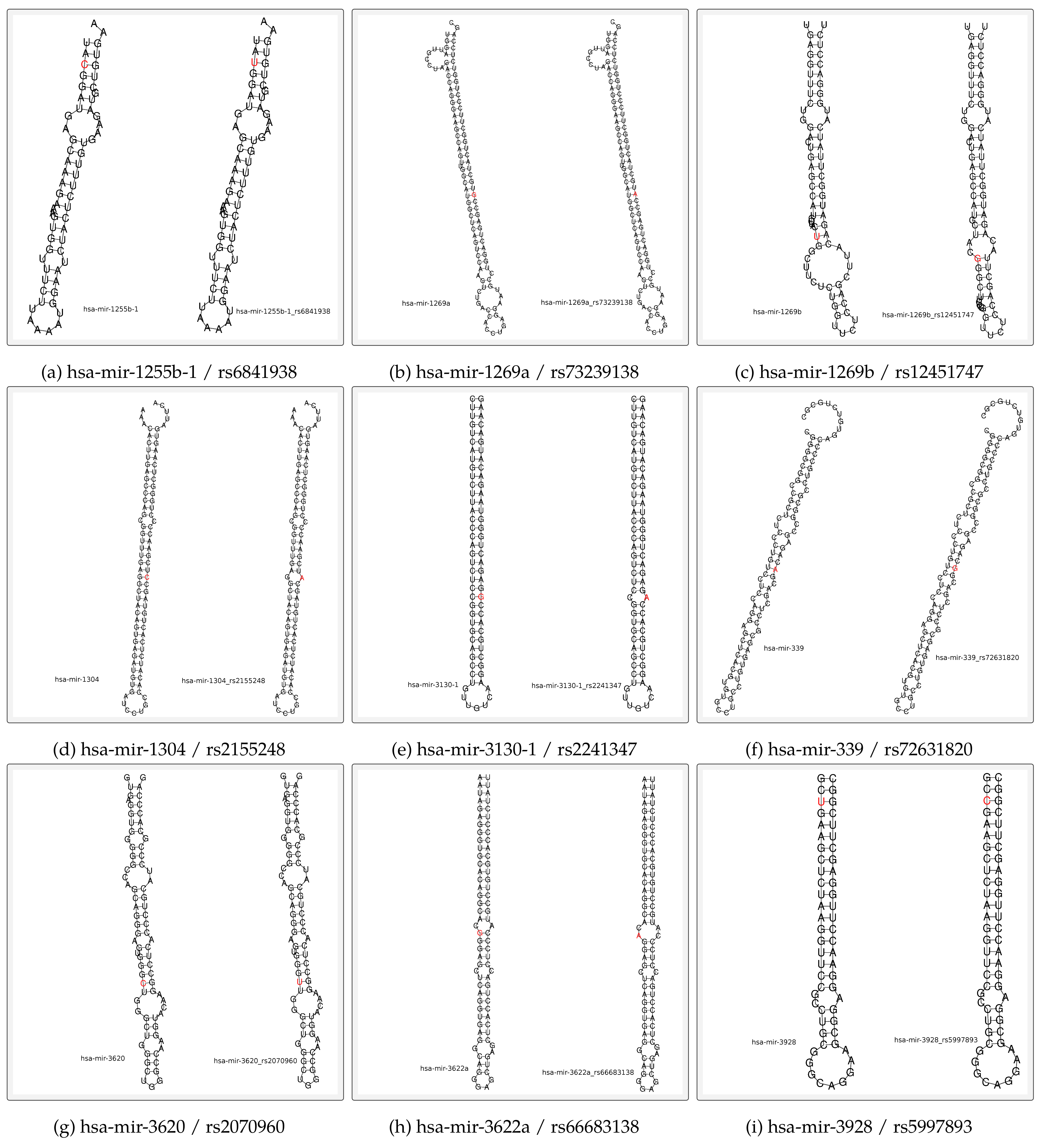
**Supplemental Figure S1 (1/4): RNAfold predictions of polymiRs secondary structures that are expressed by heterozygous samples**

**Supplemental Figure S1 (2/4): RNAfold predictions of polymiRs secondary structures that are expressed by heterozygous samples**

**
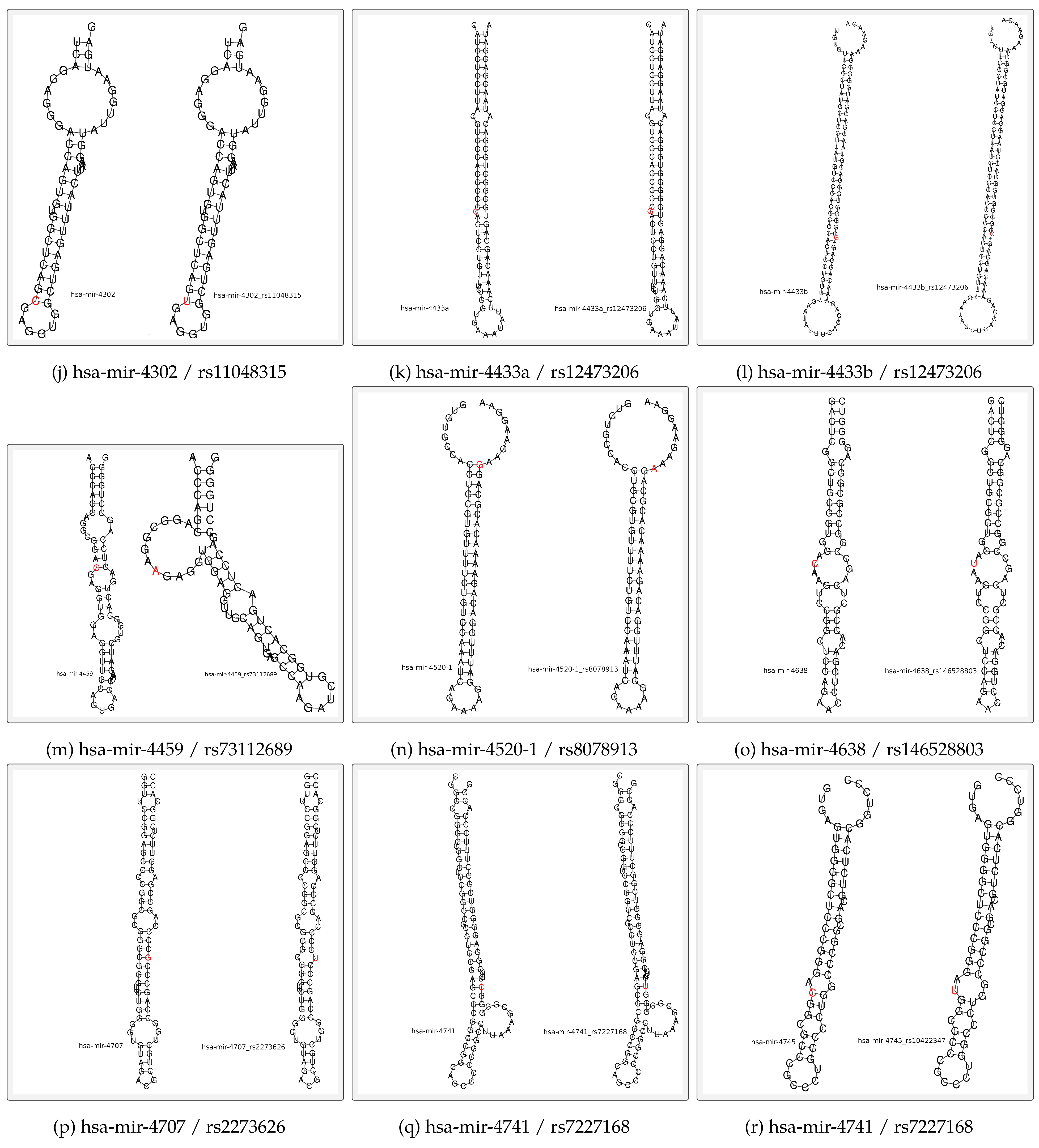
**

**Supplemental Figure S1 (3/4): RNAfold predictions of polymiRs secondary structures that are expressed by heterozygous samples**

**
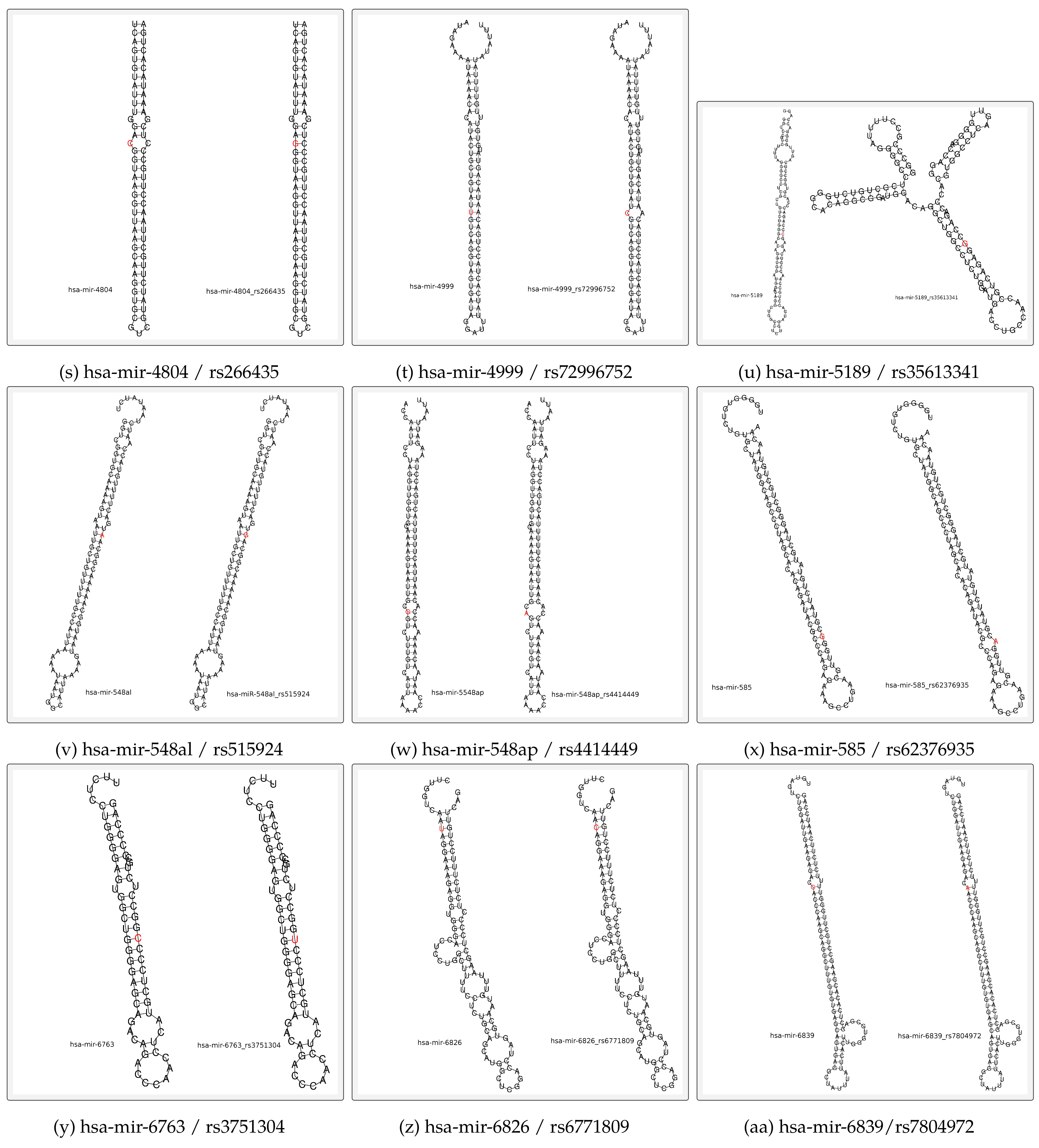
**

**Su****pplemental Figure S1 (4/4): RNAfold predictions of polymiRs secondary structures that are expressed by heterozygous samples**


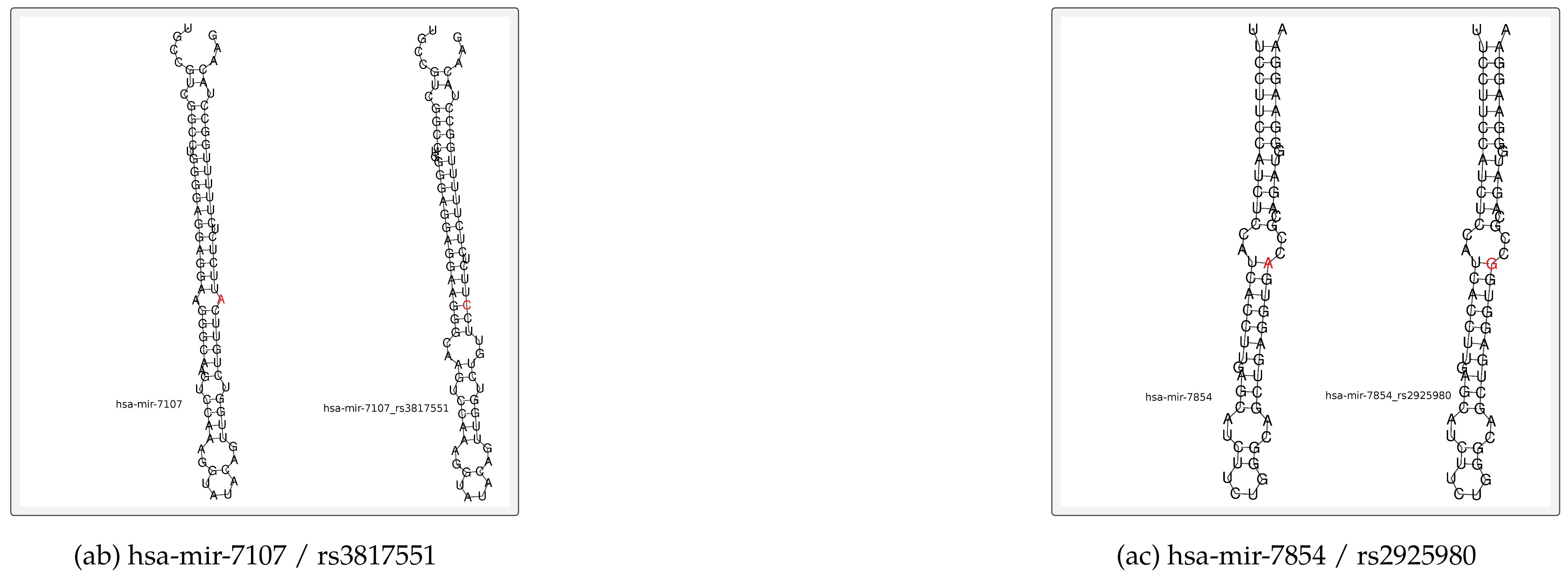

Supplement: Supplemental Material [file supp_069708.118_Supplemental_Fig_S1.docx]
