# Supplementary figures and images for: OPTIMIR, a novel algorithm for integrating available genome-wide genotype data into miRNA sequence alignment analysis
Source: RNA. 2019 Jun;25(6):657–68. doi: 10.1261/rna.069708.118 (PMC6521604; doi:10.1261/rna.069708.118)

**Supplemental Figure S2 : Library preparation workflow**


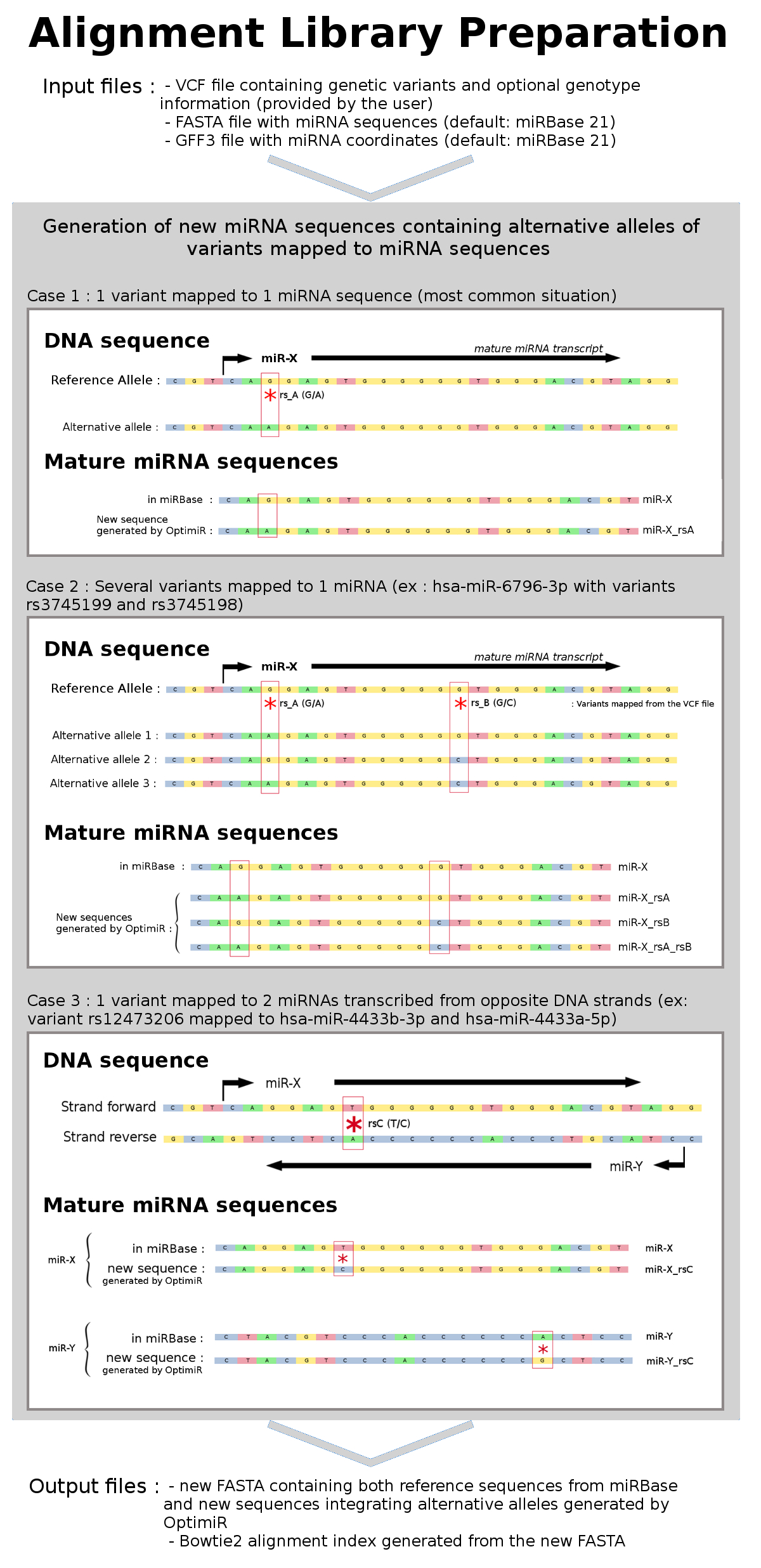

Supplement: Supplemental Material [file supp_069708.118_Supplemental_Fig_S2.docx]
